# Configuring a Powered Knee and Ankle Prosthesis for Transfemoral Amputees within Five Specific Ambulation Modes

**DOI:** 10.1371/journal.pone.0099387

**Published:** 2014-06-10

**Authors:** Ann M. Simon, Kimberly A. Ingraham, Nicholas P. Fey, Suzanne B. Finucane, Robert D. Lipschutz, Aaron J. Young, Levi J. Hargrove

**Affiliations:** 1 Center for Bionic Medicine, Rehabilitation Institute of Chicago, Chicago, Illinois, United States of America; 2 Department of Physical Medicine and Rehabilitation, Northwestern University, Chicago, Illinois, United States of America; 3 Department of Biomedical Engineering, Northwestern University, Evanston, Illinois, United States of America; University of California, Irvine, United States of America

## Abstract

Lower limb prostheses that can generate net positive mechanical work may restore more ambulation modes to amputees. However, configuration of these devices imposes an additional burden on clinicians relative to conventional prostheses; devices for transfemoral amputees that require configuration of both a knee and an ankle joint are especially challenging. In this paper, we present an approach to configuring such powered devices. We developed modified intrinsic control strategies—which mimic the behavior of biological joints, depend on instantaneous loads within the prosthesis, or set impedance based on values from previous states, as well as a set of starting configuration parameters. We developed tables that include a list of desired clinical gait kinematics and the parameter modifications necessary to alter them. Our approach was implemented for a powered knee and ankle prosthesis in five ambulation modes (level-ground walking, ramp ascent/descent, and stair ascent/descent). The strategies and set of starting configuration parameters were developed using data from three individuals with unilateral transfemoral amputations who had previous experience using the device; this approach was then tested on three novice unilateral transfemoral amputees. Only 17% of the total number of parameters (i.e., 24 of the 140) had to be independently adjusted for each novice user to achieve all five ambulation modes and the initial accommodation period (i.e., time to configure the device for all modes) was reduced by 56%, to 5 hours or less. This approach and subsequent reduction in configuration time may help translate powered prostheses into a viable clinical option where amputees can more quickly appreciate the benefits such devices can provide.

## Introduction

As lower limb prosthetic options advance, so too does the promise of increased mobility for the more than 600,000 individuals in the United States who live with a major lower limb amputation [1]. Microprocessor-controlled passive prosthetic knees are one of the most advanced classes of prostheses currently available to transfemoral amputees. These devices use onboard sensors to adjust their mechanical response during swing and stance phases of movement. Although clinical comparisons of these devices with their predecessors (non-microprocessor-controlled passive knees) have been mixed, microprocessor-controlled passive devices have been shown to reduce energy expenditure during walking [2]. However, these passive devices cannot provide net positive mechanical work to aid the user during locomotion.

A more recent generation of microprocessor-controlled prostheses that are mechanically powered have the potential to provide amputees with near physiological power at the knee and/or ankle. A few powered prostheses, such as the Ossur Power Knee [3] and BiOM Ankle System [4], are currently on the market while others are in development [5], [6]. Powered devices use a variety of control methods such as finite state machines in combination with an impedance-based model [7], artificial reflexes [8], or complementary motion estimation [9]. Regardless of the control method, these prostheses have the ability to assist amputees in performing more demanding tasks or activities that require net positive mechanical work, such as climbing a flight of stairs, ascending a steep ramp, or standing up from a seated position. Thus far, the additional power provided by these devices has allowed transfemoral and transtibial amputees to walk and climb stairs with kinematics and kinetics that more closely resemble those of non-amputees [10]–[13].

The rapid advances in prosthetic hardware and the potential for amputees to achieve additional ambulation modes (e.g., stair ascent using a reciprocal gait) imposes an increased burden on the clinicians who must configure these devices for daily use. Aside from standard socket and alignment adjustments, microprocessor-controlled passive prosthetic knees require more complex configuration than non-microprocessor-controlled passive devices. For example, the prosthetist must manually set damping characteristics within a larger number of gait cycle phases, and these devices also allow adjustments in the transition to swing phase. Since powered microprocessor-controlled prostheses use motors to provide virtual stiffness and damping characteristics in all phases of the gait cycle, their configuration is inherently more complex—configuration possibilities for some of these devices are essentially endless [14]. While this flexibility may be advantageous for optimizing control during research and development, it can easily become a challenge in the clinic, especially when fitting combined knee and ankle devices to transfemoral amputees. Recommended starting points for specific configuration parameters as well as information on which parameters to modify to provide the most benefit for a given user would simplify clinical configuration of powered knee and ankle prostheses.

Information regarding configuration processes for powered lower limb prostheses is limited. Previous research with a powered knee and ankle prosthesis designed by Vanderbilt University [5], has relied on empirical tuning of joint impedance by combining visual inspection of kinematics with feedback from one amputee user (e.g., [11]). Earlier research with the BiOM Ankle System describes adjusting ankle stiffness, damping, and power delivery such that the angle of the prosthetic ankle at toe off and net positive mechanical work matched average biological ankle data [15]. The amount of time required to configure these powered devices is unclear. In addition, little is known about how configuration parameters generalize across individuals of various amputation levels, body weights, and activity levels.

The goal of this study was to develop a configuration approach for a powered knee and ankle prosthesis over five ambulation modes including level-ground walking, ramp ascent/descent and stair ascent/descent to reduce the initial accommodation period across various modes. This approach consisted of several novel stance phase intrinsic control strategies in an impedance-based control architecture as well as a set of starting configuration parameters to provide comfortable ambulation across a range of transfemoral amputees. Finally, we developed a list of desired clinical gait kinematics and the configuration parameter modifications required to improve them. We hypothesized that our approach would reduce the number of variables to be empirically tuned and hence reduce the time required to configure the device (i.e., the initial accommodation period) across the five modes of ambulation for novice powered prosthesis users.

## Methods

### A. Powered Prosthesis Control

**Figure 1:**
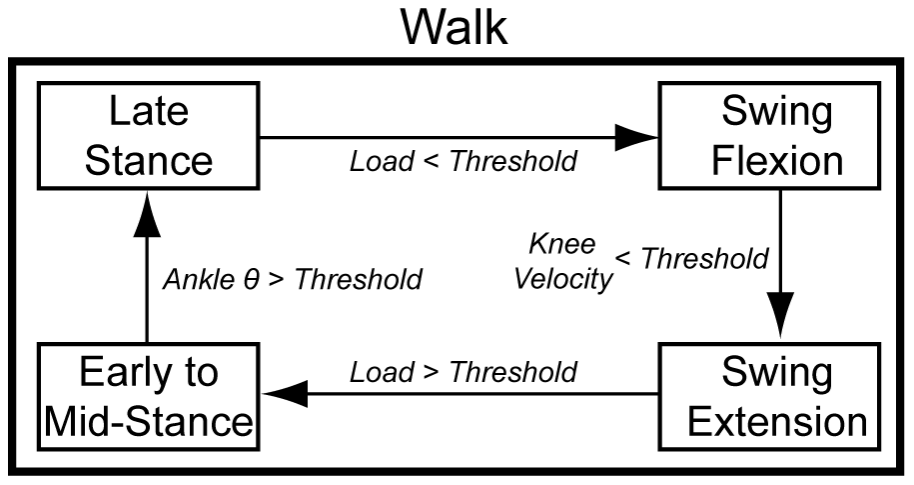
Diagram of finite state machine for level ground walking.

The powered knee and ankle prosthesis was designed by researchers at Vanderbilt University [5] and comprised two brushless DC motors with belt-driven transmissions that provided power to the knee and ankle. The prosthesis included a custom carbon fiber foot, a foot shell, and a shoe; total prosthesis mass was 4.5 kg. The device could provide up to 90 Nm of torque at the knee and 100 Nm at the ankle. The device was controlled using an impedance-based model that generated torque commands, 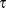, for the knee and ankle joints according to Eq. (1):
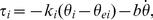
(1)where 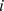 corresponded to the knee or ankle, 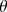 was the joint angle where negative values represented knee flexion and ankle plantarflexion and positive values represented knee extension and ankle dorsiflexion, and 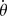 was the joint angular velocity. The three impedance parameters of each joint were stiffness, 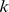, equilibrium angle, 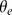, and damping coefficient, 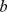, which were modified using a finite state machine for each ambulation mode. The state machine architectures were similar to previous designs [5], [12], [16], but only included four states (Fig. 1). The stance phase was divided into two states: early to mid-stance and late stance, and the swing phase was divided into two states: swing flexion and swing extension. Four transitions (between early to mid-stance, late stance, swing flexion, and swing extension states) were triggered based on mechanical sensor thresholds (Fig. 1). Across 5 ambulation modes (level-ground walking, ramp ascent/descent, stair ascent/descent), a total of 140 parameters (120 configuration parameters comprised of 3 impedance parameters for 2 joints in 4 states per mode and 20 transitions) could be modified.

Across ambulation modes, 60% of the impedance parameters were set to constant values within each state. Five modified intrinsic control strategies were implemented to adjust the remaining impedance parameters. The five strategies included (1) basing impedance parameters on set values from the previous state; mimicking biological joint responses (i.e., modifying joint impedance as a function of (2) ankle angle); modifying joint impedance as a function of (3) knee angle; or allowing users to control the rate of power generation or dissipation (i.e., modifying joint impedance as a function of (4) decreasing or (5) increasing axial force in the prosthesis). Brief descriptions of these control strategies are provided below including the potential benefits of these strategies in comparison to using constant impedance parameters. Additional details regarding strategies implemented during walking can be found in [17], [18].

#### (1) Constant Impedance Based on Previous State

To smooth the transition between states and reduce the number of independently tuned parameters, some impedance parameters were set to either the constant value from the previous state or instantaneous joint angle upon entering the state.

- Level-ground walking, ramp ascent, and ramp descent during early to mid-stance; set 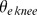 to the instantaneous knee angle at heel strike (i.e., the knee angle upon entering early to mid-stance) to provide a smooth weight acceptance phase.
- Stair descent during swing flexion; set 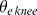 to the equilibrium angle in the previous state and 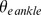 to the instantaneous ankle angle at toe off to provide a smooth transition to swing phase.

#### (2) Impedance as a Function of Ankle Angle

Ankle stiffness, 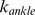, was modified as a function of ankle angle based on studies designed to identify the ankle stiffness of able-bodied subjects during walking [17]:
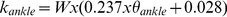
(2)where 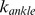 represented joint angular stiffness (Nm/deg), and 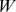 represented the user's body mass (kg). Ankle stiffness was constrained to always increase during periods of ankle dorsiflexion throughout the stance phase in level-ground walking, ramp ascent, and ramp descent. Configuring ankle stiffness based on Eq. (2) meant that this parameter did not have to be empirically tuned across users for these modes and states.

#### (3) Impedance as a Function of Knee Angle

Knee equilibrium angle, 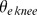, was modified as function of knee angle, 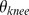:
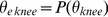
(3)where 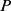 was a constant less than 1, corresponding to a percentage of the instantaneous knee angle. Knee equilibrium angle “followed” the current knee angle dictated by the constant, 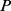, with moderate knee stiffness and damping values. Eq. (3) modulated knee equilibrium angle during early to mid-stance of ramp descent and the entire stance phase during stair descent. While Eq. (3) did not reduce the number of empirically tuned parameters (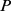 was tuned to each subject), it did provide appropriate stance phase support during controlled knee flexion for ramp and stair descent.

#### (4) Impedance as a Function of Decreasing Prosthesis Load

Joint impedance, 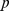, was modulated as a linear function of axial force, 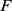, within the prosthesis [18]:
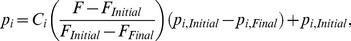
(4)where 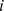 was an index corresponding to the knee or ankle, and 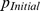 and 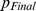 were desired initial and final values of the impedance parameter in a state. 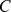 scaled the rate at which an impedance parameter changed as a function of load. For all applications of Eq. (4) in which 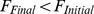, the value of 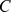 was constrained to be greater than or equal to 1, and 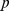 was constrained to be between 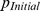 and 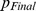. 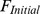 was set to the instantaneous force upon entering the state (e.g., 100% body weight) and 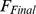 was set to 10% body weight. This strategy was applied during the following modes and states:

- Level-ground walking, ramp ascent, and ramp descent during late stance; modulated 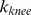 (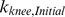 was set to the knee stiffness in the early through mid-stance and 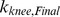 was set to the knee stiffness in swing flexion), 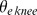 (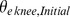 was set to the knee equilibrium angle in early through mid-stance and 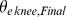 was set to the knee equilibrium angle in swing flexion), and 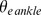 (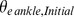 was set to the ankle equilibrium angle in the early to mid-stance and 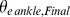 was set to −12 degrees) for reduced knee stiffness, knee swing initiation, and powered plantarflexion as force decreased.
- Stair ascent during late stance; modulated changes to 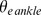 (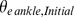 was set to the ankle equilibrium angle in early through mid-stance and 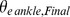 was set to −20 degrees) for powered plantarflexion as force decreased.

While Eq. (4) does introduce more variables, the majority of them are set a priori (e.g., 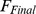), set instantaneously upon entering the state (e.g., 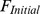), or set to corresponding configuration parameters in the previous (e.g., 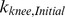) or subsequent states (e.g., 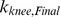). While the configuration parameters in Eq. (4) may need to be empirically tuned across users (e.g., 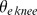 during swing flexion), the parameters within Eq. (4) do not. Incorporating Eq. (4) into the control of the prosthesis allowed users to walk over level ground and up and down ramps at their desired speed (e.g., the faster the user transferred weight off the prosthesis, the faster the impedance parameters changed). Contrary to previous empirical tuning studies for variable-speed walking [5], modifications to users' walking speed did not require modifications in configuration parameters [18].

#### (5) Impedance as a Function of Increasing Prosthesis Load

Eq. (4) was also used to modify impedance as axial force increased. For all applications of Eq. (4) in which 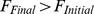, 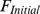 was set to the instantaneous force upon entering the state (e.g., 10% body weight) and 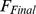 was set to 100% body weight. This strategy was applied during the following modes and states:

- Stair ascent during stance; modulated 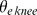 across entire stance phase (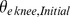 was set to knee equilibrium angle in in swing extension and 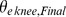 was set to 5 degrees) and 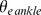 in early through mid-stance (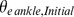 was set to ankle equilibrium angle in swing extension and 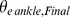 was set to 0 degrees) for knee and ankle power generation, respectively, as force increased.
- Stair descent during early through mid-stance; modulated changes to 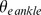 (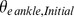 was set to ankle equilibrium angle in the swing extension and 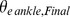 was set to 0 degrees) for controlled ankle dorsiflexion as force increased.

Eq. (4) allowed users to ascend stairs at their desired speed (e.g., the faster the user transferred weight onto the prosthesis, the faster the equilibrium angles changed resulting in faster power generation at the knee and ankle); modifications in stair ambulation speed did not require modifications in configuration parameters.

### B. Experimental Protocol

#### (1) User Characteristics

**Figure 2:**
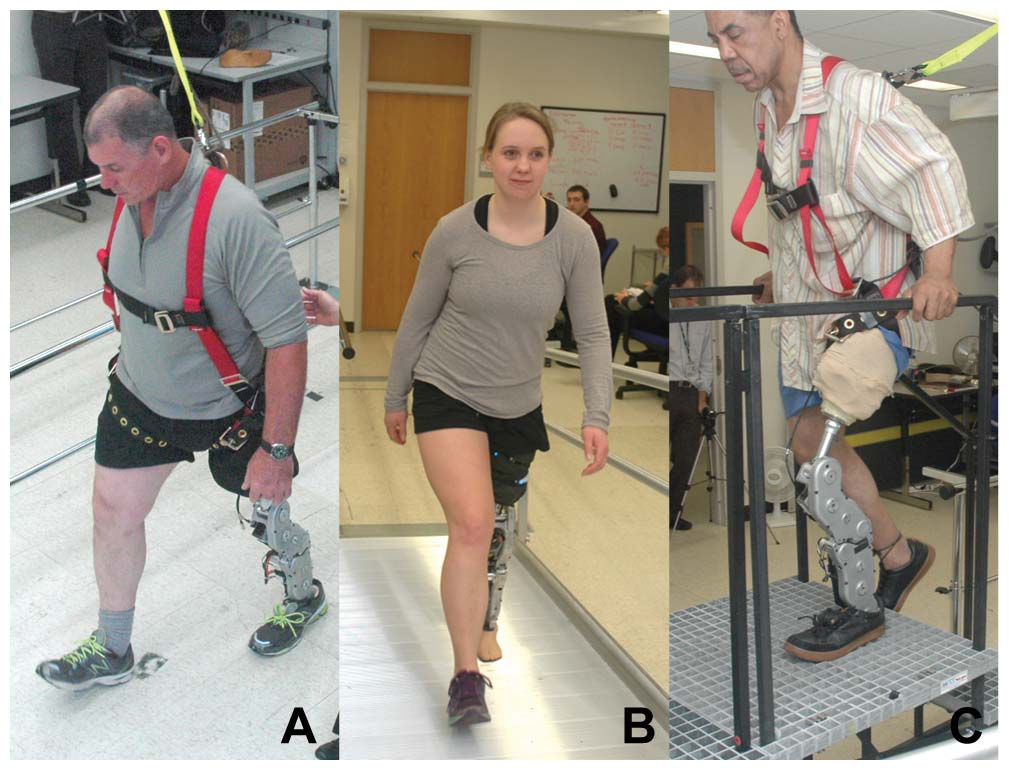
Users (A) walking, (B) ascending a ramp, and (C) climbing stairs using the powered prosthesis.

Five individuals with transfemoral amputations (TF1–5) and one individual with a knee disarticulation (TF6) (Table 1) participated in the study. All individuals provided written informed consent to a protocol approved by the Northwestern University Institutional Review Board and written informed consent (as outlined in PLOS consent form) to publish these case details. We confirmed that all users' prosthetic setups could accommodate the total build height of the powered knee and ankle prosthesis (49.2 cm) below their socket. This achieved a level pelvis and symmetric total system height but did not address potential discrepancies between the prosthesis and contralateral side knee center heights due to the fixed distance from knee joint center to ankle joint center of the powered prosthesis. Three users (TF1, TF2 and TF3) had previous experience (a minimum of 10 hours) walking on the powered prosthesis and were considered experienced users for the purposes of this study. The remaining three users (TF4, TF5 and TF6) had never walked on the powered device and were considered novice users. All users were fit with the prosthesis (Fig. 2) by a certified prosthetist and instructed by a licensed therapist.

**Table 1:** **User Characteristics.**

*User*	*Gender*	*Age (years)*	*Time Post-Amputation (years)*	*Etiology*	*Weight (kg)*	*Height (m)*	*Mobility Level* a	*Prescribed Knee Prosthesis*	*Previous Powered Prosthesis Experience*
TF1	Male	56	44	Left Traumatic	83	1.80	K3	Mauch	10 hrs
TF2	Male	64	38	Right Traumatic	86	1.75	K3	C-Leg	20 hrs
TF3	Female	22	6	Left Sarcoma	52	1.60	K4	C-Leg	20 hrs
TF4	Male	51	38	Left Traumatic	111	1.86	K3	3R80	None
TF5	Female	46	23	Right Traumatic	62	1.65	K3	C-Leg	None
TF6	Male	28	16	Left Sarcoma	86	1.87	K4	Hydraulic	None

a Medicare functional mobility levels: K3, unlimited community ambulator with the potential for ambulation with variable cadence; K4, active adult or athlete with the potential for ambulation exhibiting high impact, stress, or energy levels.

#### (2) Development of a Generic State Machine

The powered prosthesis was empirically tuned for the three experienced users [7]. Similar to standard prosthetic configuration and training, a set of clinical goals for ambulation (Table 2) guided the tuning of each mode. Amputee-specific and not amputee-specific validated clinical outcome measures (e.g., timed walk tests [19] and the Amputee Mobility Predictor with Prosthesis [20]), are likely not sensitive enough to differentiate the effects of subtle prosthetic parameter changes on gait characteristics. Therefore clinical prosthetic configuration and tuning remains a subjective process wherein the experience of the prosthetist and therapist are relied upon to determine whether prosthetic configuration changes lead to improvements or deteriorations in the user's biomechanics. As such, for this study, configuration parameters were modified for each user using a combination of visual inspection of kinematics and feedback from the user, prosthetist, and therapist. Prosthetic knee and ankle kinematic data were monitored in real time and compared to non-amputee gait [21], [22]. Clinicians observed the users' gait in both the sagittal and coronal planes and used the set of clinical goals for ambulation to discern whether configuration parameter adjustments or modifications to the user's technique (e.g., verbal/tactile cues for improved step length, balance or posture adjustments, etc.) were indicated. If a parameter modification led to an improvement in gait, the change was preserved and recorded; if not, the parameter was reset and another parameter was altered in order to achieve the desired outcome. This guided parameter sweep continued until all clinical goals for ambulation were achieved, the user was comfortable ambulating on the powered prosthesis for all five ambulation modes, and the therapist and prosthetist were satisfied with the user's performance. The tuning of each mode is described in further detail below. While adjustments were being made, users wore a harness or a gait belt for safety.

**Table 2:** **Clinical Goals for Ambulation.**

*Goal*	*Feedback*	*Walk*	*Ramp Ascent*	*Ramp Descent*	*Stair Ascent*	*Stair Descent*
Controlled weight acceptance onto the prosthesis	Clinician	✓	✓	✓	✓	✓
Appropriate amount and timing of ankle plantarflexion	Clinician, User,	✓	✓	✓	✓	✓
Appropriate knee power (generation/absorption) during stance	Clinician, User			✓	✓	✓
Appropriate amount of swing clearance	Clinician	✓	✓	✓	✓	✓
Appropriate step length	Clinician	✓	✓	✓		
Ambulate at desired speed	Clinician, User	✓	✓	✓	✓	✓
Ambulate without upper extremity support	Clinician	✓	✓			
Ambulate with minimal upper extremity support	Clinician			✓	✓	✓
Reciprocal stepping without cueing and appropriate foot placement	Clinician		✓	✓	✓	✓

Each session began with users walking inside a set of parallel bars until they were comfortable with the device; they then continued to walk outside the bars. We tuned the prosthesis for level-ground walking until users could ambulate without upper extremity support, had controlled weight acceptance onto the prosthesis (e.g., the prosthetic leg was extended ready to accept their weight at heel strike, and plantarflexion was controlled during early stance), appropriate swing clearance, and could ambulate at their desired speed (e.g., had appropriately timed powered plantarflexion in late stance (Eq. (4)), and appropriately timed swing extension).

All users had ascended stairs with their prescribed prosthesis in a “step-to” manner (leading with their contralateral side). Thus, special care was given to allow users a gradual accommodation to stair ascent using a reciprocal gait with the powered prosthesis. Users began by ascending only the first step of the staircase, leading with the prosthesis. The initial power delivery of the knee and ankle were reduced during one-stair ascent, which allowed each user to become familiar with this ambulation mode. A therapist worked with each user to practice proper foot placement, a slightly forward trunk lean, and a smooth weight shift onto the prosthesis. As users became comfortable with the one step task, power at the knee and ankle were gradually increased (i.e., increasing knee and ankle stiffness during stance) until they felt an appropriate amount of assistance. Users were instructed to allow the prosthesis to assist them up the stairs and to use a light touch on the handrails, rather than using their arms to pull them up the stairs. Users then progressed to ascending the entire four step staircase using a reciprocal gait. In addition to providing sufficient prosthetic joint power (Eq. (4)), we ensured appropriate prosthetic foot swing clearance and a flat foot placement at heel strike during stair ascent. Once the user was comfortable ascending the staircase, they practiced performing seamless transitions from walking mode to stair ascent mode.

During stair descent, users were instructed to use a reciprocal gait and to “ride” the prosthesis (i.e., allow controlled stance phase knee flexion and use the knee and ankle support) with a light touch on the handrails. One experienced user descended stairs with his prescribed prosthesis in a “step-to” manner (leading with their prosthetic side) so additional care was taken to teach him how to descend stairs using reciprocal gait. Parameters were adjusted to provide flat foot placement at heel strike (Eq. (4)). Adjustments were made to also provide sufficient knee support and control for the user's preferred speed of descent (Eq. (3)).

For ramp mode, users were instructed to ascend with a slightly forward trunk lean and to descend by “riding” the prosthesis—i.e., allow stance phase knee flexion—while keeping an even stride length. Further instructions were given to use the knee and ankle for support and to lightly touch the handrails, if needed. As for level-ground walking, we ensured users had smooth weight transfer onto the prosthesis (e.g., the prosthetic leg was extended ready to accept their weight at heel strike and the ankle plantarflexed in a controlled manner during early stance), appropriate swing clearance, and could ambulate at their desired speed (e.g., appropriately timed powered plantarflexion in late stance (Eq. (4)) and appropriately timed swing extension). For ramp descent, additional parameter adjustments were made to provide sufficient knee support and control for the user's preferred speed of descent (Eq. (3)).

Three to five sessions of empirical tuning were needed for each user in order to configure the device for safe, comfortable ambulation in all modes. These sessions totaled 6.5, 15.5, and 11.0 hours for TF1, TF2, and TF3, respectively. The empirically determined configuration parameters for each experienced user were then compiled. We found that roughly 15% of all available configuration parameters varied across the three users, and that the changes, motivated by the list of clinical goals for ambulation, were focused on a set of desired kinematic characteristics (Table 3).

**Table 3:** **Kinematic Characteristics and Corresponding Impedance Parameters.**

*Modes*	*Desired Kinematics*	*State*	*Knee Parameters*			*Ankle Parameters*		
			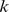	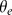	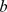	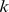	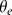	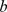
**Walk, Ramp Ascent**	Control plantar flexion at heel strike	Early to mid-stance						✓
	Provide adequate swing clearance	Swing flexion		✓		✓	✓	✓
	Position knee and ankle for heel strike	Swing extension	✓		✓	✓	✓	✓
**Ramp Descent**	Control speed of descent	Early to mid-stance	✓	✓	✓			
	Control plantar flexion at heel strike	Early to mid-stance						✓
	Change speed of knee flexion	Late stance			✓			
	Provide adequate swing clearance	Swing flexion				✓		✓
	Position knee and ankle for heel strike	Swing extension	✓		✓	✓		
**Stair Ascent**	Provide power for assistance up stairs	Early to mid-stance and late stance	✓					✓
	Provide adequate stair clearance	Swing flexion		✓				
	Control flat foot placement on stair	Swing extension		✓			✓	
**Stair Descent**	Control speed of descent	Early to mid-stance and late stance	✓	✓	✓			
	Control flat foot placement on stair	Early to mid-stance and late stance				✓		✓
	Provide adequate stair clearance	Swing flexion						✓

A generic state machine was populated with the 85% of parameters that were independent of subject. These data formed the basis of a generalized set of impedance parameters that served as a starting point in subsequent configuration sessions for all users. For the parameters that varied across subjects, nominal values—in most cases, average values—were assigned. Other parameters could be directly normalized to the subject's body weight (e.g., 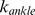 during stance, Eq. (2)), or set equal to a subject's body weight (e.g., 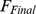 in Eq. (4) for increasing prosthesis load). In addition, we found that several parameter modifications in one mode could frequently be propagated through other modes. For example, all parameter changes made for level-ground walking were also made for ramp ascent. Based on these data, a set of detailed tuning tables were created to enable quick and efficient parameter changes within and across modes (e.g., Table 3).

#### (3) Testing the Configuration Approach with Experienced and Novice Users

Each user, regardless of experience level, began with the generic state machine. At the start of every session, the body weight of the user wearing the powered prosthesis was recorded and corresponding parameters were updated. Users were trained following the same therapy protocol outlined above, however, instead of a parameter sweep for each mode, parameters were modified in accordance with the targeted clinical goals for ambulation (Table 2) and tuning tables (Table 3). One novice user descended stairs with his prescribed prosthesis in a “step-to” manner (leading with their prosthetic side) and two novice users descended ramps with their prescribed prosthesis in a sideways “step-to” manner (leading with their prosthetic side) so additional care was taken to teach them how to descend stairs and ramps using reciprocal gait. Parameter adjustments were made until the goals were achieved, the user was comfortable ambulating on the powered prosthesis for all five ambulation modes, and the therapist and prosthetist were satisfied with the user's performance.

### C. Data Analysis

Modifications to the generic state machine as well as the number of hours each user required to comfortably achieve the five modes of ambulation were documented. Prosthetic knee and ankle kinematics of the novice users were generated and compared to those of experienced users. Data were sampled at 500 Hz and segmented across multiple strides from heel strike to heel strike.

## Results

### A. Configuring the Device for Experienced and Novice Users

The generic state machine and tuning tables, along with our modified stance phase intrinsic control, dramatically reduced the number of parameter changes necessary to configure the powered prosthesis for both experienced and novice users. Using the novel configuration approach, the three experienced users collectively required modifications to 21 independent configuration parameters of the possible 140 parameters and the novice users required modifications to 24 parameters. The parameters of the triggers between states did not change across users. On average, individual users required only 2–3 changes per mode (Table 4).

**Table 4:** **Number of Unique Independent Tuning Parameter Changes Per User.**

	*Experienced Users*				*Novice Users*			
*Mode*	TF1	TF2	TF3	Total	TF4	TF5	TF6	Total
Walk	2	1	1	3	1	9	5	9
Ramp ascent	0	0	0	0	0	0	0	0
Ramp descent	3	4	4	8	1	3	4	6
Stair ascent	3	2	3	5	3	2	4	5
Stair descent	1	0	4	5	2	1	3	4
All modes	9	7	12	21	7	15	16	24

All users, regardless of experience level, were able to walk using the starting values of the generic state machine, but changes were made to improve joint kinematics and user comfort. Table 5 outlines the parameter values by mode and phase and show which parameters were independent of users, which varied across users, which were constant within a state, and which varied with a state. Key parameter changes for walking were made to the knee equilibrium angle during swing flexion to provide an appropriate amount of prosthetic foot clearance. Several parameter changes were unique to novice users, such as modification of knee and ankle stiffness, damping, and equilibrium angle parameters during swing extension in order to prepare the knee and ankle for heel strike. Additionally, stance-phase ankle stiffness, damping, and equilibrium angle parameters were changed for some novice users to facilitate smooth and comfortable forward progression from heel strike through mid-stance.

**Table 5:** **Final Impedance Parameter Values by Mode and Phase for All Users.**

*Modes*	*Phase*	*Knee Parameters*			*Ankle Parameters*		
		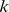 *(Nm/deg)*	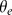 *(deg)*	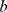 *(Ns/deg)*	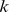 *(Nm/deg)*	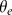 *(deg)*	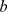 *(Ns/deg)*
**Walk, Ramp Ascent**	Early to mid-stance	3	Angle at state entry	0.25	*Eq (2), 2.5 to 7*	0	**0.25–0.3**
	Late stance	*Eq (4), 3 to 0.4*	*Eq (4), 0 to 50–70*	0.05	*Eq (2), 2.5 to 7*	***Eq (4), 0 to −12***	0.1
	Swing flexion	0.4	**50–70**	0.05	**2.5–5**	**0–2.5**	**0.1–0.25**
	Swing extension	**0.45–0.57**	0	**0.08–0.1**	**2–5**	**0–2.5**	**0.05–0.25**
**Ramp Descent**	Early to mid-stance	**2–3**	***Eq (3), P = 0.95–0.98***	**0.15–0.25**	*Eq (2), 2.5 to 7*	0	**0.25–0.3**
	Late stance	*Eq (4), 2–3 to 1*	*Eq (4), Final value in previous state to 50*	**0.05–0.1**	*Eq (2), 2.5 to 7*	*Eq (4), 0 to −12*	0.1
	Swing flexion	1	50	0.05	**1.5–2.5**	0	**0.1–0.3**
	Swing extension	**0.4–0.55**	0	**0.05–0.1**	**1.5–2**	0	0.15
**Stair Ascent**	Early to mid-stance	**2–4**	*Eq (4), 50–60 to 5*	0.15	5	*Eq (4), 10–20 to 0*	**0.1–0.25**
	Late stance	Value in previous state	*Eq (4), 50–60 to 5*	0.15	5	*Eq (4), 0 to −20*	0.1–0.25
	Swing flexion	1.2	**90–120**	0.15	1.5	20	0.2
	Swing extension	0.5	**50–60**	0.1	1.5	**10–20**	0.2
**Stair Descent**	Early to mid-stance	**0.4–1.5**	***Eq (3), P = 0.85–0.98***	**0.08–0.28**	**1.5–2**	*Eq (4), −20 to 0*	**0.08–0.2**
	Late stance	Value in previous state	*Eq (3), P = 0.85–0.98*	Value in previous state	Value in previous state	0	Value in previous state
	Swing flexion	1	Final value in previous state	0.05	1.5	Angle at state entry	**0.2–0.25**
	Swing extension	0.45	5	0.1	1.5	−20	0.2

Independent parameters that varied across users are indicated in bold and the range of values across all users is provided.

Parameters whose values varied within a state are indicated in italics and the governing equation is referenced.

During stair ascent, knee and ankle power was increased by increasing knee stiffness and decreasing ankle damping. Four of the six users (2 experienced, 2 novice) required changes to the ankle equilibrium angle during swing extension of stair ascent in order to achieve flat foot placement on the step (Table 5). After these parameter modifications, all users were able to comfortably ascend a four-step staircase using a reciprocal gait with minimal upper extremity support.

Four users (2 experienced, 2 novice) were able to descend ramps and stairs using the starting values from the generic state machine. For the remaining two users, modifications to knee stiffness, damping, and equilibrium parameters during stance phase of ramp and stair descent were required for them to comfortably ride the prosthesis down. Initial knee parameters made it too difficult for one user to bend the knee (i.e., knee was too stiff) and too easy for another user (i.e., knee too loose). These changes were expected as not all of the users performed these tasks with their prescribed, passive prosthesis using a reciprocal gait. Furthermore, changes to knee stiffness and damping during swing extension of ramp descent were made to appropriately extend the knee prior to heel contact.

### B. Accommodation Time

With fewer configuration parameters to modify, the amount of time required to configure the prosthesis for a new user was drastically reduced. Starting from the generic state machine, each experienced user was able to achieve safe and comfortable navigation of walking, ramps, and stairs in one 2.5 hour session. Each novice user required one or two sessions to completely configure the prosthesis, totaling 2.5, 5.0, and 4.5 hours for TF4, TF5, and TF6, respectively.

### C. Joint Angles and Powers

**Figure 3:**
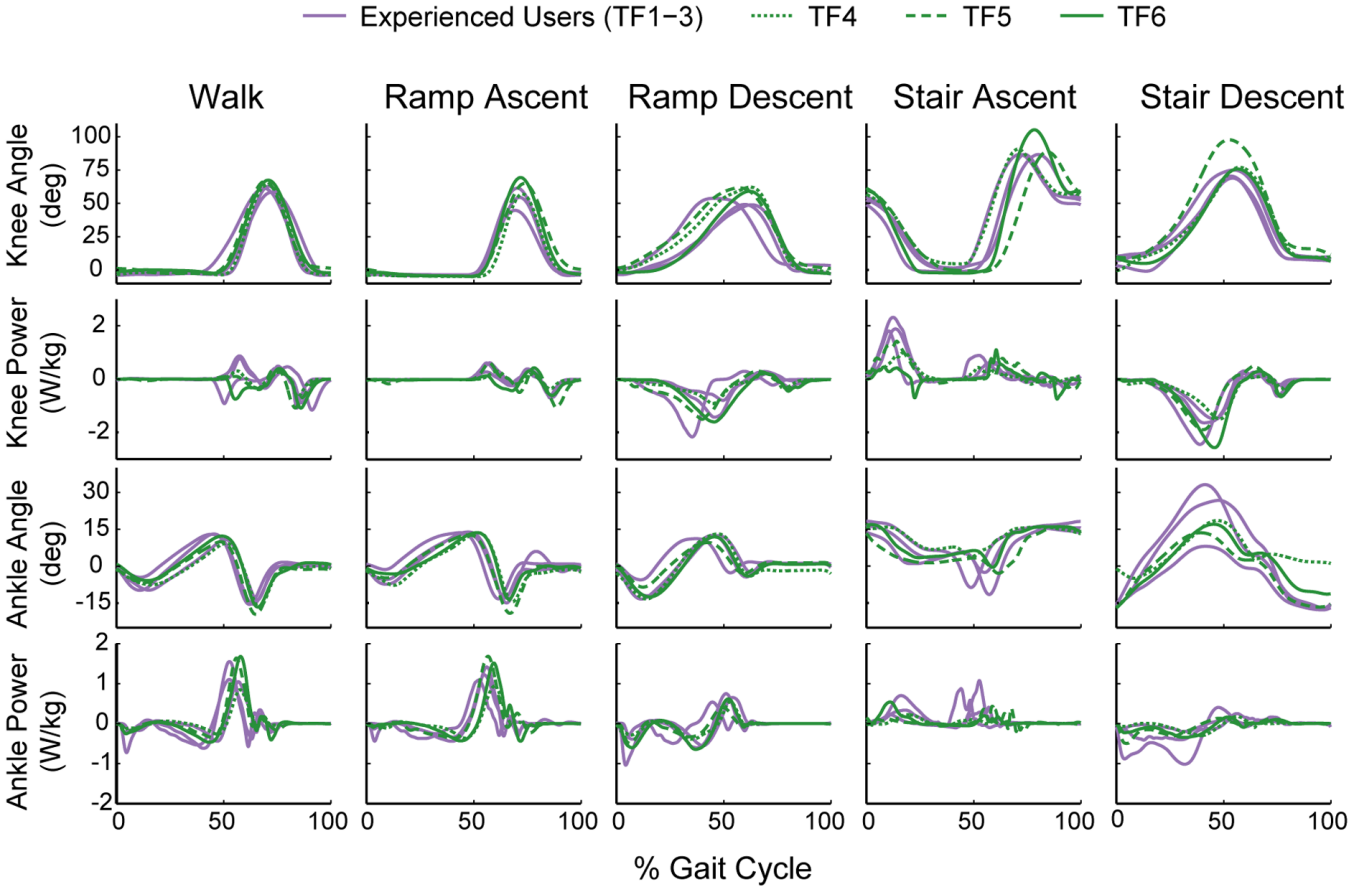
Average prosthetic knee and ankle joint kinematics and kinetics for each user. Joint powers are normalized by the mass of the user wearing the powered prosthesis.

Across ambulation modes, the kinematic and kinetic profiles of the prosthetic knee and ankle between the experienced and novice users were very similar (Fig. 3). All users were able to ascend and descend ramps and stairs using a reciprocal gait. Similar to non-amputees, prosthetic ankle kinematics and kinetics during walking and ramp ascent demonstrate controlled plantarflexion during early stance, controlled dorsiflexion through mid-stance, and powered plantarflexion during late stance. Ramp and stair descent kinematics demonstrate users' ability to use stance phase knee flexion to “ride” the prosthesis during these modes. For stair ascent, prosthetic knee kinematics were also similar to non-amputees, and a smooth development of knee power was developed in early to mid-stance (Fig. 3).

## Discussion

This study details the successful development, implementation, and evaluation of a novel configuration approach for transfemoral amputees learning to walk on a powered knee and ankle prosthesis. With the use of modified stance phase intrinsic control strategies compared with [11] and tuning tables that link desired changes in kinematics to specific parameter changes, only 24 of the 140 configuration parameters across five modes of ambulation needed tuning for novice users. On average, a total of 13 parameters were modified for novice users across all five modes.

The intention of the modified stance-phase control strategies was to increase the interaction each user had with the prosthesis (the implementations of Eqs. (2)–(4)), which was supported by a small number of within-subject parameter adjustments (i.e., increasing the generalizability of the modified control strategies across users). Setting impedance as a function of decreasing prosthesis load and ankle stiffness as a function of ankle angle has been shown to generalize well across speed during level-ground walking and ramp ascent, with no additional tuning parameter changes necessary to produce gait mechanics similar to non-amputee gait [18]. Extending these and the additional strategies to the other modes such as ramp descent and stair climbing may also allow users moderate variation in their gait without additional parameter changes. For example, setting impedance parameters based on values from previous states or instantaneous joint angles will allow users a much smoother transition from stance to swing phase during ramp descent than if empirically tuned values were chosen. If users chose to “ride” the prosthesis down to a deeper or shallower knee angle (e.g., they transferred their weight onto the prosthesis faster or slower), this strategy could automatically accommodate for those changes. Without the addition of these stance-phase control strategies, the timing of state transitions most likely would have become more important. By blending the impedance parameter changes across states and varying their rate of change based on user-modulated signals (e.g., axial load in the prosthesis) the timing of state transitions is more forgiving.

Using the powered prosthesis, ambulation modes that could not be performed with their prescribed prostheses were restored to these individuals. For example, all users were able to ascend stairs with a reciprocal gait, an activity none of them had performed since before their respective amputations. Even while users were learning to perform these new ambulation activities, the configuration strategy reduced overall prosthesis configuration time for both experienced users and novice users. Using the configuration strategy reduced the average amount of time required to configure the prosthesis for three novice users by 56% compared to the amount of time previously required to empirically tune the prosthesis for three experienced users. As expected, novice users required more time to initially tune the prosthesis but still were able to achieve comfortable ambulation in all five modes in 5 hours or less. This reduction in configuration complexity and configuration time benefits both clinicians and amputees. The initial customization of settings may help users build confidence in the device, potentially resulting in more natural walking in a shorter amount of time. All our users were able to quickly experience the benefits that a powered knee and ankle prosthesis can provide them across multiple ambulation modes.

Across all modes, the resulting kinematic and kinetic data from the prosthesis side were similar in both amplitude and timing to non-amputee gait [21], [23] and to that of one transfemoral amputee whose powered knee and ankle prosthesis was empirically tuned [5], [12], [16]. However, a specific discrepancy with non-amputee gait is that stance phase knee flexion of the prosthesis was not observed during walking or ramp ascent. The majority of our users were accustomed to extending their hip at heel strike and locking their prescribed, passive, prosthetic knee into extension during stance. While the impedance-based approach can allow for stance phase knee flexion [5], preliminary testing showed that when stance phase knee flexion was configured, users fought to override the powered prosthesis' response in favor of fully extended knee. While this compensatory movement may be overcome through more therapy, it was not the goal of this study and therefore we did not configure the device or train users to perform this typical feature of non-amputee gait. However, all six users did display many kinematic and kinetic features only available with a powered prosthesis, including powered ankle push off at near physiological levels during the late stance phase of walking [23] and powered knee extension during stair ascent [24].

While the impedance parameter values used to configure this powered knee and ankle prosthesis and achieve similar gait biomechanics most likely would be different for a different device, the stance-phase intrinsic control strategies can be applied to other powered prosthetic or orthotic devices. Table 5 is included in this study as a reference to allow comparison of the behavior between states, modes and across users. The range of stiffness and damping coefficient values used was determined by the mechanical capabilities of the device [5]. Further analysis of the results suggests that the number of independent parameter changes could likely be further reduced. Small changes in equilibrium angle (e.g., values ranging from 0 to 2.5 across users for ankle equilibrium during swing phase of walking and ramp ascent) most likely did not cause noticeable changes in kinematics across users and so may not need to be configured for each subject. Determining relationships between tuned stiffness and damping coefficients (e.g., damping ratios) of one joint or between the knee and ankle joint may assist in more quickly configuring the device for novice users. The powered prosthesis used in this study had a fixed distance from the knee joint center to ankle joint center. For four users (two experienced, two novice), this distance was shorter than their contralateral side while for the other two users (one experienced, one novice) this distance was taller than their contralateral side. This discrepancy did not present a major limitation in this study; all users were able to successfully use the powered prosthesis for all activities. The next generation of the powered knee and ankle prosthesis is modular, which allows for better prosthetic alignment and may help tease out the interplay between knee and ankle joint impedance values.

As more transfemoral amputees are fit with the powered knee and ankle prosthesis, we will continue to update the configuration strategy, tuning tables, and generic state machine parameters. The current configuration strategy was developed from three experienced users and tested with three novice users. Testing users with a wider variety of relevant characteristics (e.g., residual limb length, weight, activity level, etc.) may allow for either further reduction in subject-specific parameter modification or uncover other correlations between configuration parameters. For example, users with similar relative residual limb lengths may require similar changes to one or more configuration parameters. Anecdotally, the two novice users who had the shortest residual limb lengths both required changes to the ankle equilibrium angle during swing extension of stair ascent to achieve flat foot placement on the stairs.

As with any prosthesis, proper clinical instruction and therapy are necessary to achieve positive outcomes. Teaching amputees how to ambulate on a powered knee and ankle prosthesis is critical. The configuration approach outlined in this study provides the foundation for this therapy to begin. We began by configuring the device while users walked on level ground and then moved to more challenging ambulation modes including stairs and ramps. We found that quickly progressing to a mode that clearly demonstrated the potential benefits of using a powered prosthesis, such as stair ascent (i.e., something that the users are unable to do with their prescribed prosthesis), peaked users' interest and enthusiasm. Once users have experience and training with the powered prosthesis, further fine tuning of configuration parameters may be necessary. This, in addition to exploring additional benefits of powered prostheses, is the focus of ongoing research.

## Conclusions

As more powered prostheses begin to enter the market, their success will be determined by several factors, including the ease of configuration, the length of the initial accommodation period, and the benefits they provide to users. Prostheses that can generate net positive mechanical work at the knee and ankle increase the number of ambulation modes restored to amputees. However, empirically modifying the prosthesis response for individual users across all ambulation modes challenges the viability of such devices in clinical settings. The modified intrinsic control strategies and set of initial configuration parameters developed here reduced the accommodation time that both experienced and novice users required to perform level-ground walking, ramp ascent/descent, and stair ascent/descent. Reducing the necessary configuration time before transfemoral amputees can independently use a powered prosthesis may allow them to more quickly appreciate the benefits such devices can provide.
